# From Microbial Communities to Distributed Computing Systems

**DOI:** 10.3389/fbioe.2020.00834

**Published:** 2020-07-22

**Authors:** Behzad D. Karkaria, Neythen J. Treloar, Chris P. Barnes, Alex J. H. Fedorec

**Affiliations:** ^1^Department of Cell and Developmental Biology, University College London, London, United Kingdom; ^2^UCL Genetics Institute, University College London, London, United Kingdom

**Keywords:** synthetic biology, microbial consortia, biological computing, multicellular systems, biotechnology

## Abstract

A distributed biological system can be defined as a system whose components are located in different subpopulations, which communicate and coordinate their actions through interpopulation messages and interactions. We see that distributed systems are pervasive in nature, performing computation across all scales, from microbial communities to a flock of birds. We often observe that information processing within communities exhibits a complexity far greater than any single organism. Synthetic biology is an area of research which aims to design and build synthetic biological machines from biological parts to perform a defined function, in a manner similar to the engineering disciplines. However, the field has reached a bottleneck in the complexity of the genetic networks that we can implement using monocultures, facing constraints from metabolic burden and genetic interference. This makes building distributed biological systems an attractive prospect for synthetic biology that would alleviate these constraints and allow us to expand the applications of our systems into areas including complex biosensing and diagnostic tools, bioprocess control and the monitoring of industrial processes. In this review we will discuss the fundamental limitations we face when engineering functionality with a monoculture, and the key areas where distributed systems can provide an advantage. We cite evidence from natural systems that support arguments in favor of distributed systems to overcome the limitations of monocultures. Following this we conduct a comprehensive overview of the synthetic communities that have been built to date, and the components that have been used. The potential computational capabilities of communities are discussed, along with some of the applications that these will be useful for. We discuss some of the challenges with building co-cultures, including the problem of competitive exclusion and maintenance of desired community composition. Finally, we assess computational frameworks currently available to aide in the design of microbial communities and identify areas where we lack the necessary tools.

## What Do We Mean by Computing With Biological Systems?

There may be as many definitions of computing as individuals willing to give one. In this review we will stick to one which is relatively general in order to allow us to draw analogy between electronic and biological computing implementations without becoming too restricted. As such, we define computing as the processing of information, to produce an output, in a manner that is encoded in a program. There are less ambiguous, yet still broad, definitions that have been used, for example to determine when a physical system computes (Horsman et al., 2014). However, our layman’s definition will suffice for this review. Although the dangers of analogizing have been well-documented (Thouless, 1953), even specifically in the field of synthetic biology (McLeod and Nerlich, 2018), we will proceed with caution.

The field of electronic computing has made great impact through the use, and evolution, of two core models: the Turing machine and the von Neumann architecture. The Turing machine defines a theoretical automaton which, according to a set of instructions, reads and writes symbols to an infinitely long tape (Turing, 1937). This model is used to demonstrate the limits of computability in what is known as the Church-Turing thesis. Although many other models of computing machines have been invented which may be faster or more efficient, none are capable of computing anything that a Turing machine cannot. The von Neumann architecture defines a “stored-program” model in which the instructions for performing computation are stored in the same way as the data on which the computation is being performed (von Neumann, 1993). This architecture includes a central processing unit (CPU) which communicates with a separate memory unit, an input and an output device. The CPU executes the instructions of the computer program and the memory stores data and instructions for the CPU. Although alternatives to both models have been explored, they remain the dominant paradigm for the design and programming of most electronic computers.

At least since Jacob and Monod (1961) famously described the *lac* operon in terms of a control system engaged in information processing, researchers have been exploring the ability of natural biological systems to compute. The engineering of *de novo* biological computation began with a demonstration of the use of DNA to solve an NP-complete Hamiltonian path problem (Adleman, 1994). Since then a large number of DNA molecular computing systems have been detailed: a molecular full-adder (Lederman et al., 2006), a small neural network (Qian et al., 2011), a non-deterministic universal Turing machine capable of solving non-deterministic polynomial (NP) time problems in polynomial time (Currin et al., 2017), all 16 two input logic gates (Siuti et al., 2013), a neural network capable of pattern recognition (Cherry and Qian, 2018), and even simple games (Macdonald et al., 2006; Pei et al., 2010). While DNA, and RNA, molecular computing is still actively being pursued, the other dominant paradigm since the advent of synthetic biology has been the use of gene regulatory networks (GRNs) within cells. Manzoni et al. (2016) provide an excellent introduction into the use of GRNs to produce Boolean logic operations; an approach which has provided some remarkable successes. However, an excellent recent perspective persuasively argues that synthetic biologists need to escape from the Boolean logic paradigm which has been so successful for electronic computation due to inherent differences between electronic circuits and biological systems (Grozinger et al., 2019).

The magnitude of the populations of cells that are used for most biotechnological applications is vast and, although our ability to engineer cells has greatly improved, the computational capabilities that we can implement in each cell is still relatively small. In computer science, these characteristics have been taken advantage of in large-scale distributed computer systems. However, it is only recently that synthetic biologists have started to move away from attempting to engineer monocultures of cells, all carrying out the same process. In this review we will introduce the current state-of-the-art in the engineering of microbial cells to compute. The limitations of the current approach of using monocultures are detailed and the concept of distributed computing is introduced as a potential solution. We review the tools available to produce distributed biological systems and suggest the current challenges to implementing such systems robustly.

## Engineering Bacteria to Compute

The first synthetic biology papers engineered a toggle switch (Gardner et al., 2000), oscillator (Elowitz and Leibler, 2000) and autoregulation (Becskei and Serrano, 2000), which can be used as fundamental components in engineering a computer (Dalchau et al., 2018): memory, clock and noise filter. Since then, the tools necessary for engineering microbes for computation have been extensively developed over the last two decades of synthetic biology research. Though some of these tools have been developed explicitly for their use in cellular computing applications, many have been used to understand natural biological systems and to develop applications such as bio-therapeutics (Ozdemir et al., 2018).

A biological switch is a bi-stable system that can be flipped between the two states. The first synthetic genetic toggle switch was built in *Escherichia coli* and was composed of two repressible promoters (Gardner et al., 2000). The product of each promoter repressed the other and chemical inducers could then be used to flip the switch between the two states. Similar switching behavior can also be achieved using transcriptional regulation (Kim et al., 2006). Multi-stable switches have been theorized (Leon et al., 2016) and implemented (Li et al., 2018) which would allow for greater than two state memory. The information storage capability of DNA has also been exploited to create cellular memory devices (Siuti et al., 2013), lasting for over 100 generations (Bonnet et al., 2012). Unlike a molecular toggle switch, DNA has the potential to encode complex sequences of data, allowing the encoding and decoding of a 5.27 megabit book (Church et al., 2012) and could extend cellular memory capabilities. However, DNA based memory is not currently switchable repeatably in the same manner as the transcriptional toggle switches.

A minimal sustained oscillator can be created with only a negative feedback loop and a time delay (Stricker et al., 2008; Hasegawa and Arita, 2013), but most biological oscillators are more complex. The repressilator (Elowitz and Leibler, 2000) was the first synthetic oscillator and consisted of a system of three cyclically inhibitory proteins. Oscillators are used in natural systems to coordinate the timing of events; the most ubiquitous example being the circadian clock, which keeps time with the day/night cycle and is found in even the most primitive organisms (Schippers and Nichols, 2014). A fast oscillator with tuneable periods as short as 13 min (Stricker et al., 2008) represents a programmable timing device that could be used to time or synchronize cellular events with high precision, such as the release of a therapeutic dose (Danino et al., 2010). The robustness of the oscillations can be improved through the addition of autoregulation (Woods et al., 2016) or a “sponge” on one of the nodes (Potvin-Trottier et al., 2016).

As previously mentioned, transcriptional networks that produce Boolean logic gates have been extensively investigated. An AND gate that integrates the output of two promoters has been implemented in single cells (Anderson et al., 2007) and later more complex logical circuits were created by wiring together multiple layers of orthogonal AND gates (Moon et al., 2012). We now have libraries of orthogonal repressor-promoter NOT gates (Stanton et al., 2014), as well as the ability to produce *de novo* CRISPR-dCas9 gates (Zhang and Voigt, 2018), that can be wired together to make complex logical functions (Nielsen et al., 2016). These advances, along with tools to reduce DNA context effects (Davis et al., 2011; Lou et al., 2012; Mutalik et al., 2013) have enabled the construction of logic circuits with a great deal complexity in common lab strains of bacteria as well as strains relevant to microbiome engineering (Taketani et al., 2020). This level of circuit complexity is only achievable through the use of automated design tools, such as Cello (Nielsen et al., 2016), which match the empirical properties of genetic logic gates to ensure they will function together.

Biological processes in cells, based on the continuous concentration of metabolites and other molecules, are naturally analog. Analog computing is more efficient, in terms of the rate of ATP consumption and the number of protein molecule required, for doing addition with a genetic circuit at the ranges of precision that are metabolically feasible in single cells (Sarpeshkar, 2014). This is due to the mathematical dependence of precision on ATP consumption and number of protein molecules differing for analog and digital genetic circuits (Sarpeshkar, 2014). Additionally, it has been shown that building the equivalent circuit using analog logic can require orders of magnitude fewer genetic parts (Qian and Winfree, 2011; Daniel et al., 2013). Analog sensing, addition, and ratiometric and power law computations were implemented using only three transcription factors (Daniel et al., 2013). This was achieved by developing tuneable positive and negative logarithm circuits and connecting them through a common output to produce more complex circuits. Perceptrons, the building blocks of artificial neural networks, produce an output that is a function of the weighted sum of multiple inputs. They have been implemented using enzymes that transduce different inputs into a common output molecule, benzoate, and a synthetic actuator circuit that sensed benzoate (Pandi et al., 2019). This was used to build a cell based adder and cell free metabolic perceptrons in which enzyme concentrations acted as weights between nodes (Pandi et al., 2019).

## Limitations of Monoculture Engineering

**FIGURE 1:**
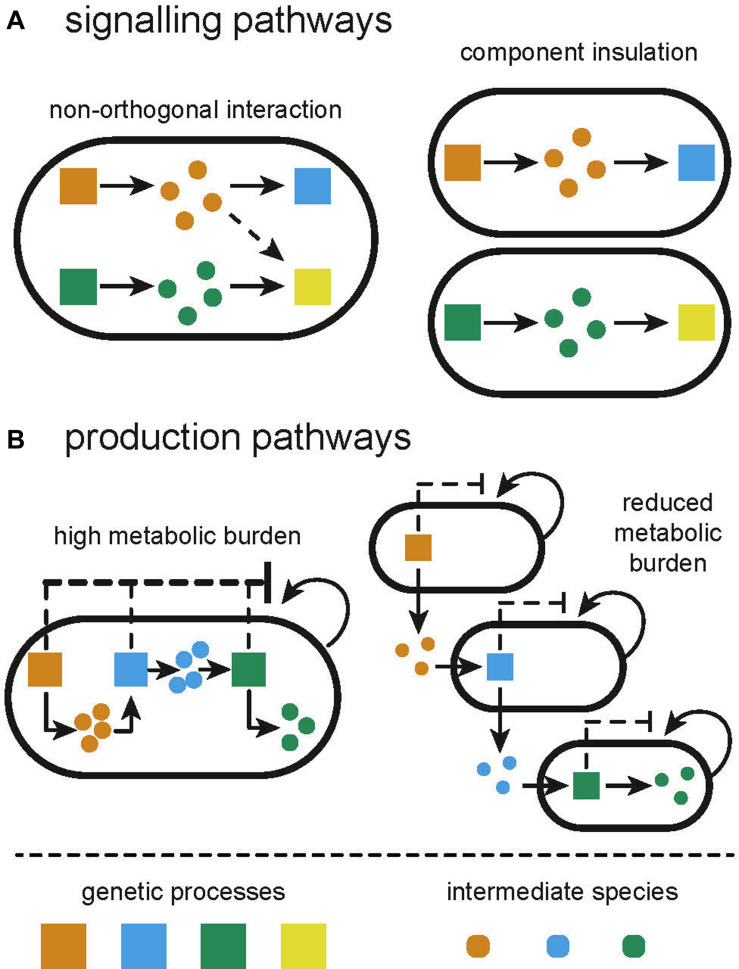
Illustration of the limitations of monocultures and how they are overcome by constructing distributed systems from multiple cell types. **(A)** Signaling pathways in monocultures can often suffer from unintended and unexpected crosstalk between processes. Compartmentalizing independent functions in separate subpopulations will prevent crosstalk. **(B)** Applications in biosynthesis suffer from high metabolic burden due to the expression of multiple heterologous processes. Division of labor between multiple subpopulations can alleviate the metabolic burden.

Components of electrical circuits are, to a great degree, insulated from one another and the environment, with interactions enabled explicitly by wiring. Heterologously expressed genetic circuits lack insulation from one another within a cell. While efforts to create subcellular compartments in prokaryotes are ongoing (Giessen and Silver, 2016), these approaches will be difficult to generalize across different circuits and applications (Menon et al., 2017). Our construction of genetic circuits in a single strain is thus limited by fundamental and interconnected concerns: non-orthogonality, retroactivity, load, and burden (Figure 1).

The library of transcriptional regulators available for the construction of genetic circuits has vastly expanded in the last two decades, particularly in model organisms such as *E. coli*. However, as we cannot directly wire one component to another, we cannot reuse components without there being a confounding interaction. Even more frustratingly, several non-identical components share similarities that lead to non-orthogonality between those components, perturbing the intended functionality of the engineered circuit (Figure 1A). As the scale of genetic circuits grows, the number of opportunities for non-orthogonal interactions grows exponentially, making it difficult to scale complexity. Efforts to circumvent this include “part-mining” to build libraries of orthogonal parts (Stanton et al., 2014) and computational design tools to incorporate known non-orthogonal interactions as part of the design process (Kylilis et al., 2018; Nguyen et al., 2019). Even the vast space of *de novo* parts enabled by CRISPR-dCas9 is limited by the number of sgRNAs that can be co-expressed before severely depleting the pool of dCas9 (Zhang and Voigt, 2018). The largest genetic circuit within a single cell, at the time of writing, consists of 55 genetic parts (Nielsen et al., 2016). In addition to such unwanted molecular interactions, sequence similarities between components can lead to mutation of genetic circuits due to homologous recombination. Libraries of parts, for example terminators, have been specifically designed that can be used together in order to circumvent this (Chen et al., 2013). Retroactivity describes a type of non-orthogonal interaction, whereby an upstream process is perturbed by a downstream species (Jayanthi and Del Vecchio, 2011). Retroactivity is common in signaling pathways with reactions that operate on different time scales, causing the accumulation of intermediate species that may interact with the upstream process (Jayanthi and Del Vecchio, 2011; Kim and Sauro, 2011; Pantoja-Hernández and Martínez-García, 2015).

The expression of genes draws from a pool of shared resources within the host. As such, the co-expression of two genes within a circuit can become coupled due to limited resource availability (Gyorgy et al., 2015). This has been compared to the load that is experienced in electrical circuits when components are placed in parallel (Carbonell-Ballestero et al., 2016). One is therefore limited in the number of components that can utilize the output from another component as their input. Since recombinant and host processes use the same resource pool, recombinant gene expression will also draw resources away from host processes causing a metabolic burden, exhibited as reduced growth rate (Glick, 1995; Figure 1B). The slower growth can encourage selection for cells which manage to lose or mutate their genetic circuit (Rugbjerg et al., 2018); strains not expressing the burdensome circuit have a competitive advantage and can outgrow the burdened population (Summers, 1991). Furthermore, metabolic burden can induce stress responses in the host, increasing mutation rates (Matic, 2013; Couto et al., 2018). Whole cell models, combining the impact of load and metabolic burden, show how changing resource availability in a host strain can produce different circuit behavior (Gorochowski et al., 2016; Boeing et al., 2018). Efforts to reduce load and metabolic burden include optimizing circuits for low copy plasmids or chromosomal integration (Lee et al., 2016), and using orthogonal ribosomes to allocate recombinant gene expression to different resource pools (Darlington et al., 2018; Boo et al., 2019). Expression of burdensome circuits can be regulated dynamically in response to population density (Gupta et al., 2017) or using promoters that are directly sensitive to burden (Ceroni et al., 2018). Mishra et al. (2014) developed a load driver for *Saccharomyces cerevisiae*, demonstrating consistent levels of expression regardless of load induced.

All of these limitations can be overcome by dividing the functionality of a circuit between subpopulations of cells, in what we will call a distributed biological system, rather than attempting to engineer a monoculture to achieve everything (Figure 1).

## From Sequential to Distributed Computing

Before discussing distributed biological systems, it is sensible to provide a short introduction to distributed computing and how it relates to other approaches to computing. In simple terms, a computer program is a set of instructions for reading, operating on, and writing data. A sequential computer processes the instructions from programs, one after the other, until the program halts. Concurrency is the execution of many programs during the same period of time, but not necessarily at the same instant. This can be achieved on a single processing unit by interleaving the instructions from multiple programs. This produces the appearance of programs running in parallel and allows the computer to respond to input from devices such as a keyboard. Although parallel and distributed computing are inherently forms of concurrent computing (many programs being run during the same time period), single processor concurrency is not true parallelism as there is still only one instruction being processed at a time.

**FIGURE 2:**
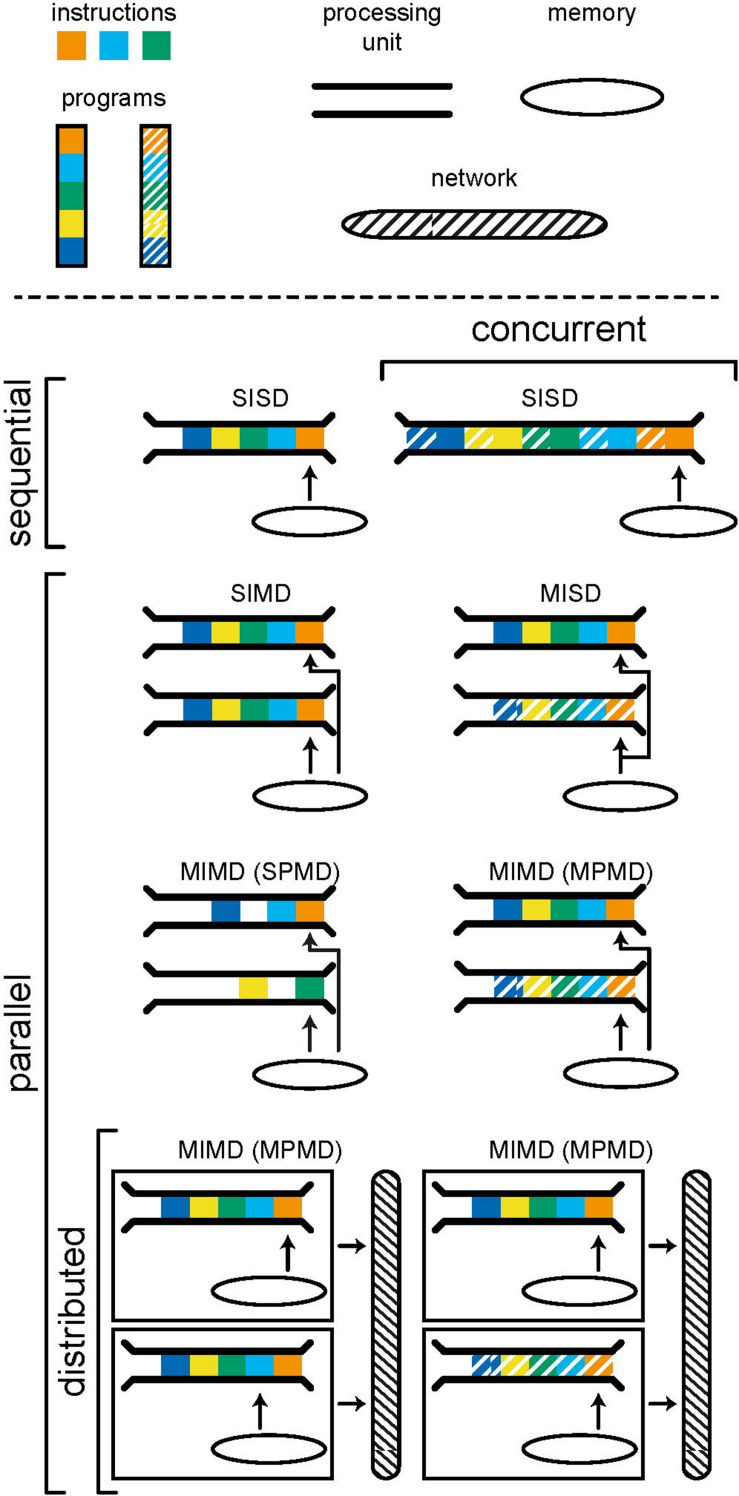
Models of computing categorized using Flynn’s taxonomy. A program consists of a number of instructions which are executed by a processing unit. A computer, or computational unit, consists of one (sequential) or more (parallel) processing units which can access data and instructions from memory. Concurrency is achieved by interweaving instructions from different programs in order to produce the appearance of parallelism. Distributed models include separate computational units which communicate by passing messages through a network. The individual computational units in a distributed system may be sequential or parallel.

Parallelism is the execution of instructions on separate processing units, simultaneously. There are many forms of parallelism and many ways of categorizing them but the most common is Flynn’s taxonomy (Flynn, 1972). This taxonomy, shown in Figure 2, uses the number of streams of instructions and data to create four categories: SISD, SIMD, MISD, and MIMD. Single-instruction single-data (SISD) corresponds to the sequential computer; one instruction is being carried out using one location in memory. In a single-instruction multiple-data (SIMD) architecture, the same operation is synchronously performed by different processor units on data from different locations in a shared memory. Graphics processors use this architecture to, for example, parallelize operations on pixels within an image. Multiple-instruction single-data (MISD) is an uncommon form of parallelism but has been employed in safety critical systems as a redundancy methodology i.e., agreement must be reached by multiple systems, exposed to the same input, for an operation to be accepted.

Multiple-instruction multiple-data (MIMD) is a form of parallelism that is now ubiquitous in modern personal computers. Here we choose to further subdivide MIMD systems to discriminate between single-program multiple-data (SPMD) and multiple-program multiple-data (MPMD). The former is a commonly used parallel programming paradigm used to speed up the runtime of a program by allowing instructions, that do not depend on results from one another, to run simultaneously on separate processing units. The limits of the speedup that can be achieved are given by Amdahl’s (fixed problem size) (Amdahl, 1967) and Gustafson’s (problem size scales with number of processors) (Gustafson, 1988) laws. It is often hard to achieve significant speedup as the requirement for independence excludes many steps within common algorithms.

MPMD is the category within which distributed systems lie. Here, different programs are run on separate processing units, accessing their own data. Distributed systems are a special case in which each processor does not have access to a shared memory and instead programs must communicate with one another through message passing. This tends to have a far higher latency (the time it takes for information to be transferred) but also higher bandwidth (the amount of information that can be transferred at once) than accessing local memory and, as such, message passing should be limited to infrequent but large transfers of data. When a distributed system is used for a common goal, there is often a control computer which assigns tasks to computers within the network and receives and synthesizes resulting data, as is common in high performance computing. Alternatively, computers within the network may have their own compulsion and the network merely allows for the sharing of resources. It is important to note that each individual computer within a distributed system can be operating in any of the categories of Flynn’s taxonomy; each computer may run the program(s) it is tasked to run sequentially or in parallel.

Models developed for describing concurrency have become the dominant models of distributed systems. Petri nets use graphs of “transitions” and “places,” analogous to instructions and memory, connected by “arcs,” to describe dynamic systems of discrete events (Petri, 1966). If the state of the places connected to a transition meet the defined requirements, the transition fires and the states of the connected places will change. Petri nets have been extensively used to model discrete chemical and biological processes (Wilkinson, 2018). The actor model consists of “actors” with their own private state (Hewitt et al., 1973). They are able to communicate only through addressed message passing and can act, concurrently, based on the messages received by sending messages, creating new actors and queuing behaviors. Finally, process calculi are a collection of algebras for modeling concurrent systems using “channels” to communicate between processes. Several variants exist that enable reasoning about, for example, systems with mobility (ambient calculus; Cardelli and Gordon, 1998), systems with changing network configuration (pi-calculus; Milner et al., 1992) and probabilistic systems (PEPA; Hillston, 1996).

Challenges specific to distributed systems relate to communication and coordination. Two foundational concepts that should be discussed here, as they have strong parallels with biological systems, are common knowledge and faulty agents. The former is detailed in an important paper in the field of distributed systems (Halpern and Moses, 1990). Individual computers within a distributed system act solely on their own local information which is learnt from their own processes and receiving messages from other computers. However, some applications require the agreement or simultaneous action of multiple computers which can only be achieved through “common knowledge,” globally known information. Halpern and Moses demonstrate that common knowledge is unattainable but detail weaker forms, such as time limited common knowledge, which allow some actions to be performed (Halpern and Moses, 1990). The problem of faulty agents is related as it concerns reaching agreements via communication of information between computers. In this scenario some of the computers in the network are faulty or malicious and, as such, the messages that they pass are unreliable. It is provably possible to reach agreement if less than one third of the network is faulty, as long as each computer knows the sender of each message it receives (Lamport et al., 1982). However, this solution requires synchronization which is not possible without common knowledge and in an asynchronous system consensus is theoretically impossible with even one faulty computer (Fischer et al., 1985), though pragmatic solutions exist (Chandra and Toueg, 1996).

There have been many attempts to draw analogies between electronic computers and biological systems as computers. The main features of a distributed system are concurrency of components, lack of a global clock, and independent failure of components (Attiya and Welch, 2004), all of which apply naturally to biological communities. From the above description of computational systems, we believe it is reasonable to consider an individual cell as a computational unit. More detailed analogies could be made, for example, between fetching an instruction and the transcription process, or performing an operation and enzymatic reactions. However, these analogies often differ depending on the abstractions that one is working on within the cell. Cells are capable of parallel processing; they are able to execute multiple tasks simultaneously. Synthetic biology to date has predominantly been undertaken using monocultures in well mixed liquids with the assumption that all cells are performing the same operation in the same environment. However, we know that heterogeneity between cells and across the environment make these systems much more analogous to distributed systems in which cells are asynchronously running the same program, exposed to different environments, alongside numerous other programs running in parallel. Further, the necessity to distribute genetic circuits across heterogenous communities of engineered cells in order to tackle the limitations of monoculture computing compels us to think of synthetic biology through the prism of distributed systems.

## Distributed Systems in Nature

Several naturally occurring biological phenomena involving cellular communities and multicellular organisms can be considered naturally occurring distributed systems. Individual cells are able to process information intracellularly and share and receive information extracellularly through, for example, the secretion of molecules.

### Bet Hedging

**FIGURE 3:**
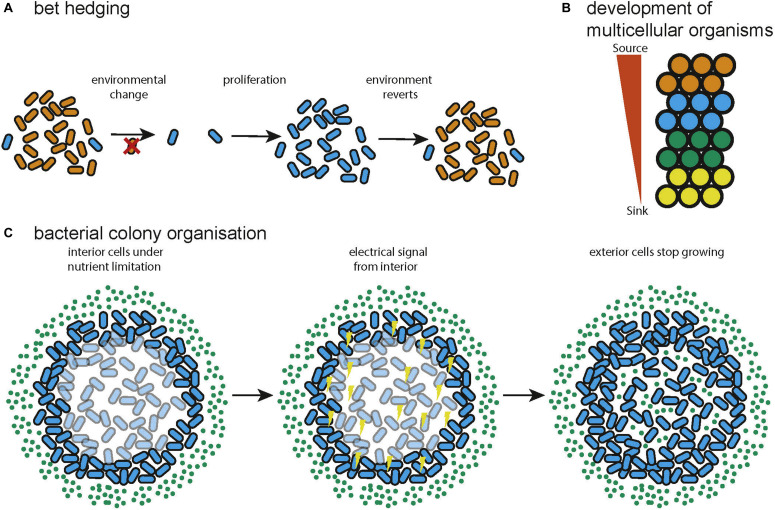
Examples of distributed systems in nature. **(A)** Bet hedging in natural bacterial communities, variation in the phenotype of the cells confers resistance to environmental change to the community (adapted from Wolf et al., 2005). **(B)** During multicellular development spatial information can be specified by gradients of diffusible molecules (adapted from Yu et al., 2009). **(C)** Bacteria can use electrical signaling to organize metabolism so that both interior and exterior cells can grow (adapted from Martinez-Corral et al., 2019).

A solution to the problem of changing environments often encountered by natural microbial communities is bet hedging. This is a strategy in which a certain percentage of a population adopt a sub-optimal state for the current environment in anticipation that the environment can change (Figure 3A). In this way the long term fitness of the community is increased by reducing the current fitness of a subset of the community. This can be entirely stochastic (Wolf et al., 2005) or biased by sensors that pick up environmental signals (Kussell, 2005). A game theoretic analysis found that switching between different losing strategies produces a winning strategy when environmental transitions cannot be sensed (a Parrondo paradox) (Wolf et al., 2005). Further, the optimal switching rates are a function of environmental properties and that diversification is favorable upon entering new environments with noisy information. It was separately shown that stochastic switching can be favored over sensing when the environment changes infrequently and that the optimal switching rates are again dependent on the properties of the environment (Kussell, 2005). Bet hedging has been demonstrated to be even more favorable when colonizing new environments, supporting the view that expanding into novel environments supports diversification (Villa Martín et al., 2019). This research shows that bacterial colonies leverage the capacity for phenotypic heterogeneity to produce a community that is optimized, according to the principles of game theory, for survival or expansion in uncertain environments. This has analogs in various forms of search and optimization algorithm, in which multiple, simple heuristics or algorithms can be explored in parallel to provide a solution (Huberman et al., 1997; Deng et al., 2012).

### Development of Multicellular Organisms

The process of development, by which a single cell becomes a morphologically complex organism composed of well-organized, heterogeneous tissue has been shown to be largely orchestrated by signaling using diffusible molecules called morphogens (Figure 3B). A theoretical model of morphogenesis was first presented by Alan Turing (Turing, 1952). This model is based on systems of multiple morphogens that react with each other and diffuse through tissue. Simulation results showed that the reaction diffusion model could correctly predict the spacing of angelfish stripe patterns (Kondo and Asai, 1995). Later work concluded that there are universal mechanisms of specifying cell spatial information, based on fields and polarities (Wolpert, 1969). A field is a group of cells that have their position specified with respect to the same set of points and polarity is the direction in which spatial information is specified. Francis Crick proposed that the fields might be produced by sources and sinks of diffusible molecules (Deuchar, 1970). This model has since been shown to be accurate for the Fgf8 morphogen in zebrafish embryos (Yu et al., 2009). A further proposed explanation of a field is that it constitutes a group of cells that are oscillating synchronously and are tightly coupled (Newman and Bhat, 2009). This could be the mechanism behind clusters of cells in the insect wing disc that progress through the cell cycle together and could also help explain how some developmental fields work over longer distances than would be possible by diffusion (Giribet, 2009). The epigenetic landscape (Waddington, 1957) for a simple regulatory network consisting of two genes has been quantified and found to behave as a potential function, with basins of attraction at the differentiated states (Wang et al., 2011). The idea of a fitness landscape has also been applied in areas such as cell signaling (Sekine et al., 2011), cell death (Zinovyev et al., 2013), and pattern formation in *Drosophila* (Lepzelter and Wang, 2008). Recent attempts to quantify spatial information during development include a demonstration that the expression level of just four gap genes can be used to specify a cell’s position with 1% uncertainty in the *Drosophila* embryo (Dubuis et al., 2013). The developmental process has been compared to mathematics (Apter and Wolpert, 1965) in which a set of basal rules is used to derive a complex structure. In this way development can be seen as the efficient compression of the spatial and cell type information required to generate a complex organism from a single cell.

### Bacterial Colony Organization

Microbiomes are diverse communities of organisms that exhibit a group metabolism (Gill et al., 2006), resistance to pathogenic invasion (Stein et al., 2013; Buffie et al., 2015) and temporal stability of community function through dynamic adaptation of community members (Coyte et al., 2015). Bacteria have developed multiple methods of exchanging information including diffusible quorum sensing molecules (Nealson and Hastings, 1979), exchanging DNA via conjugation (Tatum and Lederberg, 1947), and even electrical communication (Prindle et al., 2015; Martinez-Corral et al., 2019). This allows the assembly and maintenance of spatial structure in a colony (Jacob et al., 2004; Ben-Jacob and Levine, 2006) and the spatial coordination of metabolism so that nutrients are shared across a community (Figure 3C; Prindle et al., 2015; Martinez-Corral et al., 2019). Additionally, using the ability of individual bacteria to sense environmental inputs and respond accordingly, bacterial colonies can adapt their spatial configuration to a changing environment, reacting to food availability or optimizing foraging. The bacteria *Paenibacillus vortex* forms highly modular colonies (Ben-Jacob et al., 1998; Ben-Jacob, 2003; Jacob et al., 2004; Ben-Jacob and Levine, 2006) in which circular modules of bacteria move around a common center. *P. vortex* can also form snake like swarms which can sense and collectively respond to input signals, for example swarming to collect multiple sources of extracellular material (Ingham and Jacob, 2008). This has also inspired an optimization algorithm called Bacterial Foraging Optimization (BFO) (Passino, 2002), a distributed optimization algorithm that mimics the foraging behavior of a colony of bacteria. BFO can be described as a variant of particle swarm optimization (Kennedy and Eberhart, 1995) that incorporates selection by using aspects of genetic algorithms (Holland, 1992). BFO has been found to be effective on real world problems such as signal estimation (Mishra, 2005) and controller optimization (Mishra and Bhende, 2007), in both cases it was found to outperform a conventional genetic algorithm in terms of convergence time or solution accuracy. Microbes can also interact through the exchange of metabolites. In this manner a bacterial community can exhibit an optimized group metabolism enabling the community to survive with minimal resources and persist in environments inhospitable to the individual microbes (Schink, 2002; Morris et al., 2013; Lau et al., 2016). Mathematical modeling suggests that syntrophy can often emerge spontaneously between pairs of microbial metabolisms (Libby et al., 2019) and much work shows that syntrophy leads to the loss of functional independence as genes are lost to minimize the energy usage of the community (Morris et al., 2012; Hillesland et al., 2014; D’Souza and Kost, 2016; McNally and Borenstein, 2018). Syntrophy commonly occurs within bacterial communities, for example during methanogenesis (Zhu et al., 2020), and the metabolic reactions within the human gut (Ruaud et al., 2020).

## Differences Between Biological and Silicon Systems

There are a few key differences between natural and man-made distributed systems that deserve highlighting. The first is the main method of communication; in a computer, components are connected by electrical wires and individual computers can communicate through wired networks which allow specific message passing. Even in wireless networks in which messages are broadcast, enough information can be attached to a message so that it is only readable by the target computer. This means that nodes in a distributed system can send messages specifically and communication networks can be set up to include arbitrary groupings of nodes. Although systems exist for the passage of messages specifically between cells (Goñi-Moreno et al., 2013), due to its ubiquity in bacteria and the relative low level of complexity quorum sensing is the dominant method of communication engineered into synthetic bacterial consortia. When communicating via quorum sensing, bacteria secrete the message in the form of a diffusible molecule. A secreted molecule sent by a cell will reach any cell within its vicinity and the requirement to read the “message” is only expression of the associated, or closely related, sensor, meaning that this is a form of broadcast communication. The rigidity of the connections between a set of computers in, for example, a local area network mean that the network can be classified as a solid network, meaning that the connectivity of the network does not change with time. This is in contrast to a community of cells, where agents can move relative to each other and agents communicate with other agents in their local area. This means that connectivity will change with time, and the community can be classified as a liquid network (Solé et al., 2019). This distinction has important implications for message passing and communication within a microbial community. For example, the “wiring problem” occurs when more than one communication channel is required within a bacterial community. Later, we discuss the current communication tools available for synthetic biologists and detail their limitations. Microbial communities are also composed of reproducing biological organisms, meaning that they are subject to selective competition and potential disruptions via mutations. This also allows natural communities to adapt to changing environments but is a fundamental challenge in synthetic biology, as will be discussed below. However, the merits of liquid networks have been investigated (Langton, 1986; Miramontes et al., 1993; Solé and Miramontes, 1995; Solé and Delgado, 1996; Vining et al., 2019) and it has been shown that liquid networks are capable of reaching a global consensus (Vining et al., 2019) and universal computation (Solé and Delgado, 1996).

A second key difference is that the great majority of electronic computers use digital memory and logic. Analog systems are often emulated on digital computers, which introduces inefficiencies in terms of power consumption and simulation time (Guo et al., 2016). Microbes are not limited to digital computation and often use analog computations to their advantage, for example the continuous responses of environmental sensors (Mannan et al., 2017) or the addition of the concentration of quorum molecules from multiple sources (Long et al., 2009). This in turn relates to how the different systems treat noise. In a digital computer variability in the output from a component is considered undesirable and, as such, error checking and correcting mechanisms are built into every level of a computer (Johnson, 1984; González et al., 1997). As detailed in the previous section natural communities, however, often harness noise in both gene expression and the genetic makeup of the community (Kussell, 2005; Wolf et al., 2005; Villa Martín et al., 2019).

## Distributed Systems in Synthetic Biology

The challenges, described previously, of non-orthogonality, load, and burden in synthetic biological systems have been confronted by the expansion of genetic parts libraries (Chen et al., 2013; Mutalik et al., 2013; Stanton et al., 2014). However, more and better parts will only push our problems further into the future. The ever-increasing capabilities of computers has enabled, and perhaps been driven by, the development of ever more demanding software. The same will happen with synthetic biology; the complexity of the systems we design will always push the limits of the parts that are available to us.

Using the principles developed over several decades of work on distributed computing and insights from research into natural biological distributed systems offers an alternative, and complementary, approach to expanding parts libraries. Distributing a system between subpopulations of cells means that we can reduce the number of parallel tasks that we are asking host cells to perform, reducing load and burden, and enabling the reuse of parts in different subpopulations without orthogonality issues.

### Available Tools for Building Distributed Synthetic Biological Systems

#### Liquid and Solid State Environments

Distributed synthetic biological systems can be assembled as liquid or solid cultures. The choice of which will be dictated by the intended application, with each choice possessing important advantages and disadvantages.

In a well-mixed liquid culture, microbial cells exist as independent entities that are free swimming. All subpopulations share approximately the same environment, offering a constant intermediary for the exchange of resources and information.

Bioreactors and microfluidic devices allow different scales of control over liquid culture environments, the choice of which plays an important role in the behavior of the populations. Over the past several years a number of low-cost bioreactors have been developed (Takahashi et al., 2015; Hoffmann et al., 2017; Steel et al., 2019). Turbidostats are a class of continuous bioreactor that maintain the culture at a constant optical density (OD) by varying the dilution rate. A turbidostat can maintain the culture in the desired growth phase indefinitely (Takahashi et al., 2015; Hoffmann et al., 2017). This is of particular interest for implementing distributed systems since gene expression profiles often differ between phases of growth (Klumpp et al., 2009). Some of these bioreactor devices can be configured to measure the output of several fluorescent proteins simultaneously and control multiple inputs dynamically (Steel et al., 2019). Dilution rate has been cited several times as a critical controllable parameter; the rate of removal of molecules from the environment can produce very different population dynamics (Balagaddé et al., 2008; Weiße et al., 2015; Yurtsev et al., 2016; Fedorec et al., 2019). As such, possessing the correct tools is important for building distributed systems in liquid culture.

Microfluidic devices have been developed that enable batch, chemostat and turbidostat cultures (Lee et al., 2011; Ullman et al., 2013). These have been used for a range of applications, such as high-throughput gene expression analysis (Lee et al., 2011; Ullman et al., 2013), elucidating the relationship between population density and antibiotic effectiveness (Karslake et al., 2016), the evolution of antimicrobial resistance (Toprak et al., 2012), and screening for fitness under different environmental conditions (Wong et al., 2018). Such devices are suited to assessing community cultures and have been applied in the microbial ecology field to understand multi-faceted interactions (Kehe et al., 2019). Microfluidic traps can be used to monitor cells in a fixed position and enable the establishment of local microenvironments while still having a regular turnover of cells and nutrients (Bennett and Hasty, 2009). Microbial traps capture some properties of solid state cultures. In some cases trap-like structures are essential for generating a critical cell density and ensuring short diffusion distances (Chen et al., 2015). Microfluidic traps can also be used to investigate the spatiotemporal dynamics of consortia and how strain interaction and signaling efficacy is affected by trap size (Alnahhas et al., 2019). A further microfluidic device has been used to investigate quorum sensing over different lengths. The effect of distance on information transmission, the robustness of a distributed genetic oscillator and mutualistic interaction between two strains was investigated (Gupta et al., 2020).

Liquid cultures provide the closest analog to a shared memory model of computing in which all processing units (the cells) have direct access to the same data (the environmental state). However, the common assumption that liquid cultures are homogenous does not stand up to scrutiny (van ’t Riet and van der Lans, 2011). Accounting for the latency in a communication network and spatial distribution of species are important characteristics to include. For example, changing flow rates in a microfluidic device can turn synchronized population oscillations into spatiotemporal traveling waves because dilution occurs non-uniformly in space (Danino et al., 2010). This suggests that, rather than using a model of shared memory that is implicit in most models of bacterial liquid cultures may be insufficient under some circumstances.

In solid state cultures, microbes will often assemble into a biofilm. Biofilms are a mass of microorganisms which adhere to a self-produced extracellular matrix (ECM) (Flemming et al., 2016). The ECM density allows for the establishment of local concentration gradients (Flemming et al., 2016) which in turn allows the formation of local niches (Poltak and Cooper, 2011). Biofilm formation itself is a form of computation through communication, invoking a pattern of gene expression to drive a developmental process (Davies et al., 1998; Sauer et al., 2002; Liu et al., 2017; Abisado et al., 2018), similar to how morphogen gradients that define cell fate are a well-characterized form of computation in mammalian cells (Christian, 2012). Members of a biofilm often experience direct cell-to-cell contact with one another, required for horizontal gene transfer through conjugation (Flemming et al., 2016; Madsen et al., 2018). Microbial ecology studies show that the community metabolic output of a biofilm is positively associated with ecological diversity (Boles et al., 2004; Poltak and Cooper, 2011). Since biofilms are often naturally diverse systems, they possess attractive characteristics for building spatially distributed systems. Studies have demonstrated control over biofilm formation in a variety of ways. Optogenetically induced gene expression systems can be used to produce defined patterns of biofilm formation (Huang et al., 2018). Quorum sensing and antimicrobial peptides can be used to generate tuneable bandpass patterns (Kong et al., 2017) or control the dispersal and colonization of biofilms in multiple subpopulations (Hong et al., 2012).

Explicit distribution of subpopulations in 3D structures may prove to be an important tool for building distributed systems in solid states. 3D-printing offers a manufacturing platform for rapid prototyping from CAD designs to three-dimensional structures (Savini and Savini, 2015). The more recent falling cost of desktop 3D-printers have made this technology an attractive option for bioengineering, replacing extruded plastics with bioinks. These are made from biocompatible materials such as hydrogels, gelatin or alginate and are designed to cross-link immediately after or during bioprinting (Gungor-Ozkerim et al., 2018). They are seeded with living cells which can be printed directly into the desired 3D conformation (Connell et al., 2013; Schaffner et al., 2017; Huang et al., 2018; Qian et al., 2019). Structures can be designed to increase mass transfer, leading to improvements in product yield (Qian et al., 2019) and distinct populations can be layered on top of one another (Lehner et al., 2017; Schmieden et al., 2018). Bacteria can be used to functionalize these materials. For example, hydrogels mixed with *Pseudomonas putida* conferred the degradation of phenol (bioremediation functionality); while improved mechanical robustness can be harnessed by mixing hydrogels with cellulose producer *Acetobacter xylinum*, suitable for biocompatible medical applications (Schaffner et al., 2017). Connell et al. (2013) demonstrated generation of “core-shell” geometries, where an internal core population can be protected from external environmental conditions by being encompassed by a distinct shell population. Such cross-species protection interactions can be observed in the oral microbiota (Marsh, 2005).

#### Modeling Approaches

The field of microbial ecology frequently uses genome scale metabolic models to infer the interactions between community members and can serve as an important guide for building large scale synthetic systems (Biggs et al., 2015). It has become common practice to build metabolic models of individual community members that can then be combined to make quantitative predictions about the metabolic dependencies and interactions. This approach has been applied to the prediction of metabolic interactions between species in the gut microbiome (Shoaie et al., 2015). Similarly, genome-scale metabolic models have been used to aide in the design of large scale communities by predicting metabolites that can be released by the producer without detriment to fitness, and conditions that encourage the establishment of stable communities (Pacheco et al., 2019). Thommes et al. (2019) used genome scale metabolic models of *E. coli* to compute feasible division of labor strategies that could arise from an initial monoculture through loss of function in genes, giving insight into possible avenues for engineering community formation. Angulo et al. (2019) demonstrated a mathematical method for identifying “driver species” in an ecological network. External control of the driver species allows the user to manipulate the state of the entire network. Approaches such as these could be a steppingstone between ecological communities and building entirely synthetic networks.

Agent-based models are a class of computational model that simulate a system of autonomous agents and their interactions. Agent-based models are effective for modeling systems with discrete elements and are useful for representing heterogenous environments and spatial distribution of species (Gorochowski, 2016). This approach has been used extensively to model formation and interactions in biofilms (Kreft et al., 1998; Lardon et al., 2011). Gro is a high-level framework for defining and simulating bacterial colony growth (Jang et al., 2012). Gro has more recently been extended to include nutrient uptake and cell-cell signaling, enabling the simulation of spatial patterning in 2D (Gutiérrez et al., 2017). Agent-based modeling frameworks DiSCUS and BactoSIM have been used to simulate conjugation processes in biofilms and how this effects the population as a whole (García and Rodríguez-Patón, 2015; Goñi-Moreno and Amos, 2015); an important form of information propagation bacterial systems. BSim 2.0 is a flexible modeling framework that can be used to simulate microbial community systems in microfluidic devices (Matyjaszkiewicz et al., 2017). The software can simulate signal expression, diffusion and response, and has been used to identify optimal microfluidic chamber design for a particular community behavior (Matyjaszkiewicz et al., 2017).

### Implemented Synthetic Biological Distributed Systems

#### Modular Logical Circuits

**FIGURE 4:**
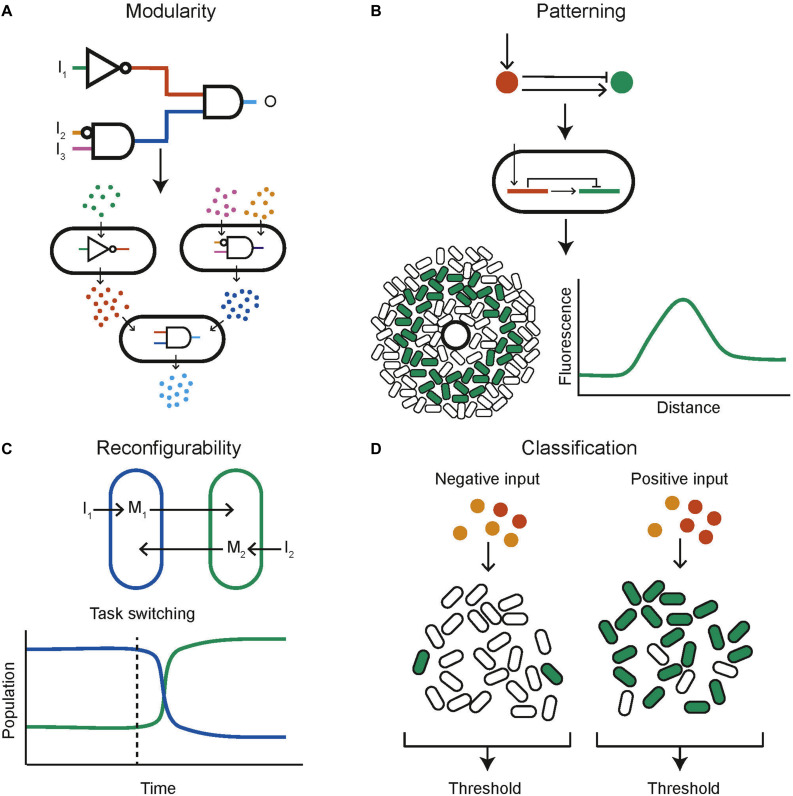
Example capabilities of computational communities. **(A)** A complex circuit can be split into modules, distributed across different populations of cells (adapted from Regot et al., 2011). **(B)** Computational methods can be used to find networks capable of stripe formation, these can be programmed into cells using genetic circuits, the expressed phenotype of each cell depends on its position relative to a source of signaling molecule (adapted from Schaerli et al., 2014). **(C)** Reconfigurability could be a key capability of biological computing. Here the composition of a bacterial community can be controlled through inducers I1 and I2. This capability could be used to task switch in a computational bacterial community (adapted from Kerner et al., 2012). **(D)** Bacterial communities are naturally applicable to complex functions such as ensemble classification (adapted from Kanakov et al., 2015).

One of the key engineering principles that synthetic biology strives to adhere to is modularity so that biological components can be recombined and interchanged to build new systems rather than needing to design full systems from scratch. A successful example within the context of synthetic biological distributed systems is the decomposition of a complex logical function into multiple subunits, each engineered within a different population of cells that communicate with each other (Figure 4A). This mirrors a common approach in electronics where two universal logic gates, for example NOR and NAND, are wired together to produce any logical function. In this manner all 16 two input logic gates have been created using bacterial colonies on agar plates, containing genetically engineered NOR gates, and communicating via diffusible molecules (Tamsir et al., 2011). A similar approach consisted of a community of yeast cells that carried out the functions AND, NIMPLIES, NOT, and IDENTITY (Regot et al., 2011). These are chemically wired together using diffusible communication molecules to produce complex functions. The output was also distributed across multiple cell types, helping to reduce wiring requirements and enabling the construction of all the two input logic gates, multiplexer and 1-bit adder with carry (Regot et al., 2011). Mathematical work into the optimal design of computational communities implementing distributed genetic logic gates given realistic constraints on the number of logic gates possible per cell and the number of orthogonal quorum molecules has been done (Al-Radhawi et al., 2020). It was found that under the assumption that any cell is limited to a maximum of seven logic gates the use of a community composed of two cell types increased the number of logic gates by 7.58-fold over the capabilities of a monoculture. Another automated design framework for the construction of user specified logical functions using DNA recombinase NOT and IDENTITY gates distributed over multiple cell types enables the design of a consortium of bacteria to perform the desired digital function (Guiziou et al., 2018). This framework was then used to build consortia capable of four input digital logic (Guiziou et al., 2019). The standard mathematical proof that any Boolean function can be decomposed into a double summation of IDENTITY and NOT logics was used to build multicellular circuits encoding the IDENTITY and NOT logic into cells and then performing sums by mixing cell cultures together (Macia et al., 2016). In this manner arbitrary logic functions can be built. A different approach using antibiotic sensitivity has been used to construct a three-bit full adder and full subtractor using *E. coli* cells with a calculator like display (Millacura et al., 2019). Combinatorial resistance was used to distinguish between different combinations of three antibiotics, then a visual output was distributed across cell types arranged in a spatial display.

#### Memory

A key component for computation is memory. Quorum sensing has been combined with a genetic toggle switch, resulting in a population level toggle switch (Kobayashi et al., 2004). A synthetic community composed of *E. coli* strains has been used to record the order, duration and timing of chemical events (Hsiao et al., 2016). Here the stochasticity of the intercellular processes was harnessed to do the encoding of memories at the population level. This facilitated functionality not possible at the level of individual cells, including recording the order and time difference between two events and the start time and pulse width of an inducer signal. A bistable switch was built across two distinct cell types, controllable by two different yeast pheromones, that switched the community between two states (Urrios et al., 2016). The simulation of a design for a flip flop memory device distributed over four populations of cells show that its function is robust to changes in parameters and that circuit behavior can be tuned by changing experimental conditions (Sardanyés et al., 2015). This design leveraged the modularity possible with a microbial community, the flip flop logical circuit was broken down into four modules that were distributed across the four cell types and the modules were wired together using diffusible molecules. Another computational investigation showed how a co-culture of two bacterial strains could be used to do associative learning, with both short- and long-term memory (Macia et al., 2017). The microbial community responds to an input (A) but not a second input (B) unless both A and B have been simultaneously present in the past. This results in a computational system that can respond differently depending on its history. Here the modularity of a co-culture was exploited again to prevent cross talk and simplify the genetic constructs required by distributing different logical components into different populations.

#### Edge Detection

A genetic light sensor and communication with diffusible signals was used to create a lawn of *E. coli* capable of edge detection (Tabor et al., 2009), an important algorithm in image recognition and artificial intelligence. An image is applied to the lawn by placing an image mask in front of a light source. Cells produce a quorum sensing molecule when not exposed to light and fluoresce when exposed to both light and the quorum signal; a combination which is only present at a light-dark interface.

#### Reconfigurable “Hardware”

Unlike electronic computers, biological systems are able to change their “hardware” depending on the task at hand by, for example dynamically controlling the constituents of a community (Figure 4C). Two independent auxotrophic *E. coli* populations have been designed so that their growth is tuneable by inducing production of amino acids (Kerner et al., 2012). Using a community of microbes that inhabit slightly different temperature niches, a temperature cycling scheme is able to dynamically tune the community (Lin et al., 2020). Methods of intrinsic community composition control can be built into cells genetically. This has been done using self-inhibition using quorum molecule signaling (Dinh et al., 2020). One strain produces an N-Acyl homoserine lactone (AHL) quorum molecule which leads to a reduction in its own growth rate when at a high concentration. This was used to control co-culture composition as the two strains of cells grew together and resulted in a 60% increase in productivity. Simulation results also show that a population of cells containing a reconfigurable logic gate that can be switched between NOR and NAND behavior (Goñi-Moreno and Amos, 2012). Furthermore, a rock-paper-scissors system of three populations of *E. coli* that cyclically inhibit one another, combined with population dependant synchronized lysis, shows the capability to cycle the community composition through the three strains (Liao et al., 2019). This was built with the intention of plasmid stability, but by using three functionally different strains a community could be built that can be cycled between different functions as required.

#### Classification

Classifiers aim to identify which category an observation belongs to. Biological classifiers have been built to identify cancer cells using miRNA (Xie et al., 2011; Mohammadi et al., 2017). A key concept in machine learning is the use of ensemble methods. These combine the output of many individual weak classifiers, which perform at least slightly better than random choice, and produce an overall output with much greater accuracy. This methodology can naturally be applied to a community of cells, where each cell contains a genetically encoded weak classifier and the overall community output is computed by combining the individual outputs of all cells (Figure 4D). This approach has been investigated *in silico*. For each data point in a training data set a heterogenous population of cells containing weak classifiers vote on the answer (Kanakov et al., 2015). The community learns as cells are stochastically pruned from the population; cells that voted incorrectly are removed with a higher probability. A multi-input classifier composed of a community of cells containing either a linear or a bell-shaped classifier was simulated and found to be able to represent practically arbitrary shapes in the input space (Kanakov et al., 2015). Other numerical results on a similar population of cells showed that complex classification problems could be tackled (Didovyk et al., 2015). In both papers, soft training, in which cells are removed with a certain probability according to their decision, outperforms hard training, in which incorrect cells are always removed and correct cells are always retained.

#### Noise Reduction

Noise in biological systems can arise due to a number of intra-cellular and environmental reasons. Although noise seems to be important to the functioning of many biological systems (Rao et al., 2002), engineered systems are required to be predictable and therefore resilient to noise. Mechanisms have been developed to reduce gene expression noise within cells. Buffer systems have been built using miRNA to degrade mRNA transcripts in a controlled manner, reducing gene expression variability at the cost of a reduced maximal expression (Strovas et al., 2014). A genetic integral feedback controller with the potential to maintain cellular system variables at desired levels despite noisy dynamics was shown to be able to control growth rate (Aoki et al., 2019). Mundt et al. (2018) dampened noise in gene expression by tuning transcription rates and the degradation rate of mRNA. Instead of implementing a complex intracellular mechanism to reduce noise, computational communities have the potential to repeat a computation over multitudes of cells and integrate the results by reaching a global consensus, vastly improving the robustness of the computation to noise inside any single cell. This is particularly important in analog computing as the continuous states of an analog computer are susceptible to small perturbations (Sarpeshkar, 2014). The global consensus problem is a fundamental problem in distributed computing (Wang et al., 2014), where multiple independent agents converge to a global consensus that is robust to failure or noise of individual agents. Modeling work on a community of agents, resembling a microbial community, that are capable of movement and local communication shows that the community is capable of solving the global consensus problem (Vining et al., 2019) and is an indication that this could be implemented in a bacterial community.

#### Patterning

Both multicellular organisms and communities of unicellular organisms have the ability to cooperate to produce spatial structures that allow them to better perform complex functions. The prime example of this phenomena is development in multicellular organism, in which cells containing identical DNA differentiate and organize themselves spatially to assemble a complex organism. Harnessing this capability could mean the realization of biological computers that can self-assemble and reproduce in a manner that is not currently possible with silicon systems. The first step in this direction was taken by engineering *E. coli* “receiver cells” which respond to a quorum molecule with a band detect activation (Basu et al., 2005). Sources of the quorum molecule could then be used to produce different patterns of fluorescence in a lawn of *E. coli*. This approach was complemented by the development of quorum molecule producing “sender” cells (Basu et al., 2004). Work has also been undertaken using senders and receivers to produce 3D patterning of mammalian cells (Carvalho et al., 2014). It is possible that sender and receiver cells could be combined to produce dynamic pattern formation in response to environmental changes. The value of using computational modeling to investigate pattern formation and design spatially structured synthetic communities has been shown (Figure 4B; Schaerli et al., 2014). Here the space of two and three-node, stripe forming networks was investigated computationally, and used to inform wet laboratory experiments. Further computational investigation using the modeling platform GRO (Jang et al., 2012; Gutiérrez et al., 2017) acts as a proof of concept for the design of bacterial colonies capable of self-assembling into spatial structures including L and T shapes (Pascalie et al., 2016). It has also been shown that synthetic communities engineered to grow with a ring shaped pattern show scale invariance, similar to natural systems (Cao et al., 2016). An artificial symmetry breaking mechanism was combined with domain specific cellular regulation resulting in artificial patterning and cell differentiation reminiscent of a simple developmental process (Nuñez et al., 2017). Interactions between motile and non-motile bacteria when grown together in a biofilm have been shown to trigger the emergence of complex patterns over time (Xiong et al., 2020).

## Challenges (And Potential Solutions) in Designing and Implementing Distributed Synthetic Biological Systems

Although several steps have been taken down the path toward distributed synthetic biological systems, some hurdles stand in the way of the paradigm becoming ubiquitous in the field.

### Building Stable Communities

**FIGURE 5:**
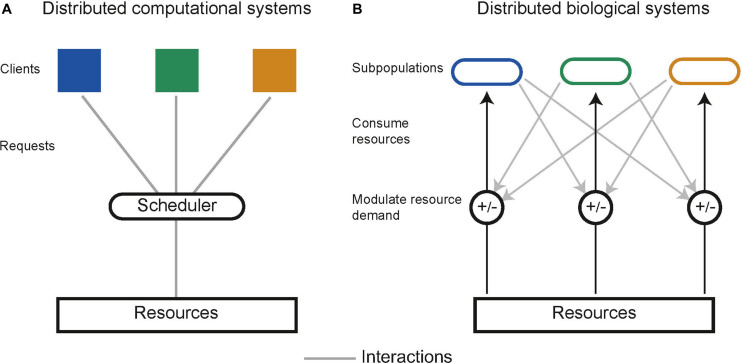
**(A)** Schematic of resource allocation in distributed computational systems. Tasks communicate with a central scheduler which in turn allocates resources to tasks. **(B)** Resource allocation in distributed biological systems is decentralized. Subpopulations communicate and interact with one another to modulate the demand for resources, which can optimally allocate resources and prevent competitive exclusion.

**FIGURE 6:**
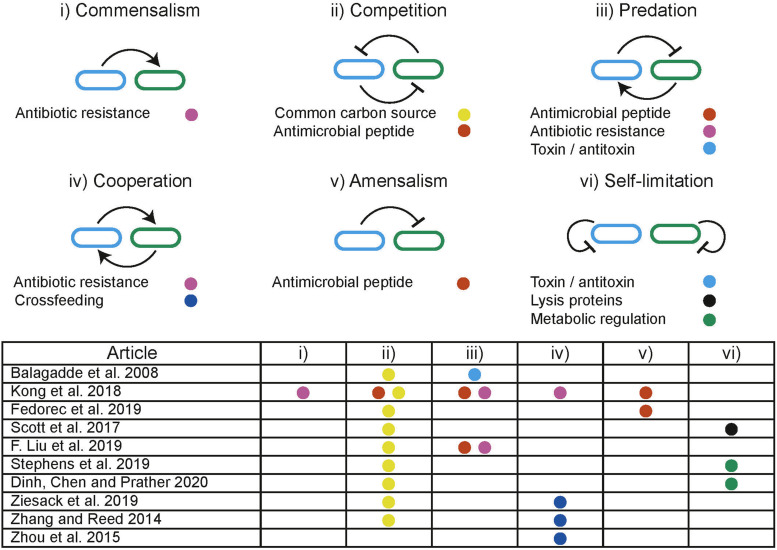
Illustration of ecological interactions that can be used to dynamically manipulate resource allocation within co-cultures. Table summarizes the ecological interactions engineered in discussed studies where the colored dots refer to the methods used to implement the interaction.

In distributed computing the execution of tasks is dependent upon limited resources such as available memory or processors. Tasks are allocated resources by central schedulers upon request, aiming to distribute resources in a “fair” and “efficient” manner while accounting for task priority (Figure 5A; Haupt, 1989). Similarly, distributed biological systems in liquid cultures are constrained by limited resources including carbon sources and essential amino acids (Jacob and Monod, 1961). Microbes tend to maximize growth, consuming the resources in a system without request. Biological systems lack a central scheduler to allocate resources fairly between subpopulations, multiple subpopulations sharing an environment therefore compete for limited resources, a single subpopulation with the highest fitness will drive the others to extinction, this is a principle known as competitive exclusion (Butler and Wolkowicz, 1985). Evidence from natural microbial systems and ecological studies shows us stability can arise through interactions between subpopulations. These interactions alter the resource demand of a subpopulation by changing its population density or metabolic activity (Figure 5B). Both cooperative and competitive interactions are important for stabilizing communities (Czárán et al., 2002; Hibbing et al., 2010; Freilich et al., 2011; Foster and Bell, 2012; Zelezniak et al., 2015; May et al., 2019). Using these principles, groups have attempted to engineer interactions as a means to ensure coexistence within synthetic microbial communities. Engineered pair-wise interactions are analogous with ecological interactions, Figure 6 summarizes studies discussed in this section, highlighting the ecological analogs that have been demonstrated synthetically, and the tools used to implement them.

Predator-prey interactions are pervasive in nature and are well-known for producing coexistence over prolonged periods. A predator has detrimental effects on the prey, while the predator is dependent upon the prey for survival. Predator-prey interactions are prevalent in natural environments and are predicted to produce limit cycle behavior indefinitely (Volterra, 1926). Planktonic predator-prey communities have been used to demonstrate long term persistence under experimental conditions and show robustness to stochastic events (Blasius et al., 2020). In synthetic biology, predator-prey interactions can be engineered between subpopulations to enable the persistence of a community that would otherwise undergo competitive exclusion. Balagaddé et al. (2008) demonstrated the use of quorum sensing (QS) coupled with toxin/antitoxin systems to implement predator-prey-like interactions. Liu et al. (2019) used modulation of a shared environment to create predator-prey dynamics. Media containing the antibiotic chloramphenicol (CM) kills the predator strain which is dependent upon the prey strain to degrade CM. In turn, the predator strain expresses IcnA, killing the prey. By providing CM exogenously, the authors created a tuneable environmental parameter that is directly involved in the social interaction.

The expression and secretion of antimicrobial peptides (AMPs) can be used to engineer amensal effects on sensitive subpopulations within a community. The signaling and AMP properties of nisin have been used with a second AMP to produce a modular system for building predatory, cooperative and competitive interactions in *Lactococcus lactis* (Kong et al., 2018). AMP microcin-V has been used with QS regulation to stabilize a two species community by engineering a single strain to have an amensal effect on another faster growing strain (Fedorec et al., 2019). Co-existence can also be achieved without engineering interactions between subpopulations. Using two strains with orthogonal QS controlled expression of lysis proteins, Scott et al. (2017) ensured that neither strain could grow beyond a threshold, thereby preventing competitive exclusion occurring through self-limitation. This effectively behaves as a block on the maximal resource occupation by any single subpopulation.

Controlling the flux of metabolites essential for growth through different pathways has been demonstrated in a monoculture using QS. The expression of a burdensome heterologous circuit was regulated, switching between “growth mode” and “production mode” in response to population density (Gupta et al., 2017). It has also been demonstrated that control over the growth rates of one strain, through modulating expression of the *ptsH* sugar transport gene, can be used to control the composition of co-cultures (Stephens et al., 2019). A similar approach was used to distribute a naringenin production pathway between two strains (Dinh et al., 2020). By using QS to self-regulate the growth of a high growth rate subpopulation combined with a low growth rate population the authors were able to generate a stable co-culture and significantly improve production yields. These examples prevent overutilization of a resource by a single strain by modulating growth directly.

Metabolic interdependencies are pervasive in microbial communities and are an important interaction that can be used to produce stable co-existence (Zelezniak et al., 2015). Interdependencies decouple the growth of a subpopulation from the limited environmental resource. Instead resources must be made available by another subpopulation in the system. Previously discussed modeling frameworks can be used to inform cross-feeding strategies and identify conditions that encourage establishment of cooperative communities (Pacheco et al., 2019). A sustainable multi-species system was generated by engineering amino acid auxotrophies and overproduction in *E. coli, Salmonella typhimurium, Bacteroides fragilis*, and *Bacteroides thetaiotaomicron* (Ziesack et al., 2019), forcing dependencies between community members. Synthetic metabolic interdependent co-cultures have been shown to undergo significant adaptation over long term co-cultures resulting in improved growth rates (Zhang and Reed, 2014). An *E. coli* – *S. cerevisiae* stabilized co-culture has been demonstrated on xylose based feed stock (Zhou et al., 2015). *E. coli* metabolizes xylose producing acetate, which is in turn used by *S. cerevisiae*. Since acetate is an inhibitor of *E. coli* growth, it is dependent on *S. cerevisiae* to remove it from the environment.

These ecological interactions manipulate the resource consumption of each subpopulations by regulating population densities and metabolic activity, providing opportunities for autonomously regulated systems. This contrasts with the centralized resource allocation commonly seen in computing. A hybrid of these approaches has been achieved through external regulation of the environment to maintain coexistence of competing. Reinforcement learning was used to train an agent that controls the supply of essential nutrients to two competing auxotrophs in a chemostat, in principle demonstrating the use of a centralized controller to regulate a biological system (Treloar et al., 2020).

### Orthogonal and Directed Communication

Quorum sensing (QS) systems are a key set of tools that enable us to engineer communications between and within subpopulations of a community. QS systems consist of one or more proteins that produce small, freely diffusible molecules. These quorum molecules bind to regulatory proteins that can activate or repress gene expression at specific promoters (Miller and Bassler, 2001). QS can be used to regulate the expression of genes in a population, but because cells broadcast to all other cells in their vicinity, each communication channel must utilize a different quorum molecule. However, in practice there are a limited number of QS systems available and even distinct QS systems may not be totally orthogonal (Grant et al., 2016). Kylilis et al. (2018) performed a comprehensive characterization of the crosstalk between several QS systems in conjunction with computational tools to identify conditions in which channels can be used simultaneously. Moreover, these tools can be used to account for and incorporate crosstalk into system design. Studies have also reduced crosstalk through rational sequence mutation (Grant et al., 2016; Scott and Hasty, 2016). Quorum quenching refers to the enzymatic degradation of quorum molecules allowing controllable degradation of QS molecules in a system. The AiiA quorum quenching enzyme and LuxI quorum molecule synthase have been used to produce oscillations in a bacterial population (Danino et al., 2010) and to introduce a negative feedback layer in a two strain oscillating system (Chen et al., 2015).

While QS is the dominant choice for engineering communication in synthetic biology, alternative channels are being developed. The γγ-butyrolactone system (derived from *Streptomyces coelicolor*) has been demonstrated E. coli to implement orthogonal signaling that can be used alongside QS (Biarnes-Carrera et al., 2018). Other signaling channels exist between different species of bacteria (Hughes and Sperandio, 2008), however, the synthetic biology field has yet to embrace these channels to the same degree as QS for controlling. Signal response mechanisms have also been observed between the host and bacteria of the human gut through polyamine compounds, highlighting the clear potential for host-community interfacing (Lopes and Sourjik, 2018).

A potential limitation of quorum sensing based approaches is that communication is non-specific and global. Cells communicate through broadcast signaling which, in contrast to the targeted information transfer afforded by electrical wires, means that each communication molecule in a bacterial community must be different in order to address different subpopulations. This acts as a constraint on the possible complexity of a distributed computation for a given number of quorum sensing molecules. In electrical engineering, circuits are only marginally constrained by the number of wires and are often optimized to minimize the number of logic gates. An analogous approach has been carried out by using an evolutionary algorithm to optimize a distributed bacterial community to reduce the number of wires (Macia and Sole, 2014). In optimized electronic circuits NOR and NAND gates are widely used. Interestingly, when optimizing for the communication constraints within a microbial community using quorum sensing, a high number of non-standard logic gates (NIMPLIES, NOT, and AND) are selected, highlighting the differences between electrical and biological computing. The optimal design of computational communities will require new tools, such as an algorithm to distribute genetic NOR gates among cell populations communicating via diffusible molecules (Al-Radhawi et al., 2020).

Other communication channels could be exploited to overcome the wiring problem. For example, the transfer of DNA between bacterial cells. The packaging and transfer of DNA messages using bacteriophage has been demonstrated in *E. coli* (Ortiz and Endy, 2012). Although this is still a broadcast approach, as in wireless networking, the amount of information that one can encode may allow selective reading of the message, for example using non-native RNA polymerases or state dependent expression. Alternatively, direct message passing has been achieved by bacterial conjugation (Goñi-Moreno et al., 2013). The sharing of conjugative plasmids has been used to design, *in silico*, a community of distributed NOR gates wired together for a population level XOR gate (Goñi-Moreno et al., 2013). Finally, electrical signaling is another potential method of communication that could allow specific message passing at a much higher speed than conjugation. Natural bacterial communities can communicate using ion channel based electrical waves similar to neurons (Prindle et al., 2015; Martinez-Corral et al., 2019) and networks of fibrous cables are used as electrical communication channels (Meysman et al., 2019). It will be exciting to see how synthetic biology can harness these behaviors over the coming years.

## Conclusion

Distributed systems are ubiquitous in modern computing, from the Internet to scientific high-performance computing. Thinking about biological systems through this lens will offer unique opportunities in the development of biological computing. A great deal of effort has been put into developing *de novo* biological systems that compute and some magnificent advances have been made. However, we are, and will remain, fundamentally limited in the systems we can build if we stick to the prevailing paradigm of engineering a single strain to do everything. The prevalence of genetically and phenotypically diverse distributed systems in nature is clear, and in this review we have highlighted some examples that we believe to be particularly relevant in the pursuit of engineering biological computation. While the prospective rewards of distributed systems cannot be overlooked, challenges in the establishment of robust and controllable distributed systems are significant but not insurmountable.

The majority of engineered biological communities demonstrated to date have focused on the establishment of co-existing populations. Building these methodologies and experimental frameworks will allow us to take the next step in focusing on exploiting communities as distributed systems. The demonstration of the advantages held by distributed systems in functionality and productivity over a monoculture will be paramount for advancing the field. The fundamental differences between microbial communities and computer networks (competition, communication and naturally analog processes) highlight opportunities for the development and advancement of the theory. These differences also present some of the greatest opportunities for functionality that is hard to achieve in digital hardware, including adaptability, self-assembly and analog information processing (Grozinger et al., 2019). Many of the competitive advantages communities have in nature are due to the ability to adapt to noisy, diverse and changing environments.

Although success has been found in overcoming these limitations and implementing familiar digital computations, focus should also be on exploiting these capabilities to build useful biological computers. Evidence indicates that the optimal organization of a bacterial computer differs from that of a digital computer (Macia and Sole, 2014). This means that new methodologies will have to be developed, extending our current capabilities of automatic circuit design in single cells (Nielsen et al., 2016) to computational communities. To realize the advantages of biological computing we will have to move away from replicating feats of electrical engineering. We envisage the biological computers will find their application niche in interfacing with biological systems. Immediately attractive applications lie in disease diagnosis through biosensing and reactive treatment through *in situ* production of biological material (Slomovic et al., 2015; Courbet et al., 2016). An open challenge to the field lies in converting the immense progress demonstrated in laboratory environments into real-world applications, validating with demonstrable improvements.

## Author Contributions

BK, NT, and AF wrote the first draft of the manuscript. All authors contributed to the conception of this review, contributed to manuscript revision, and read and approved the submitted version.

## Conflict of Interest

The authors declare that the research was conducted in the absence of any commercial or financial relationships that could be construed as a potential conflict of interest.
